# A rare right perirenal hemangioma mimicking a retroperitoneal “malignant lesion”: Diagnostic and procedural challenges of an extra-renal vascular mass

**DOI:** 10.1016/j.radcr.2026.09.005

**Published:** 2026-09-18

**Authors:** Allan Xu, Ahmed Abdulrahim, Elias Salloum, Omar Shwaiki, Wei Lue Tong

**Affiliations:** a USF Health Morsani College of Medicine, University of South Florida, 560 Channelside Drive, Tampa, FL 33602, USA; b Department of Pathology, H. Lee Moffitt Cancer Center and Research Institute, 12902 USF Magnolia Drive, Tampa, FL 33612, USA; c Department of Diagnostic Imaging and Interventional Radiology, H. Lee Moffitt Cancer Center and Research Institute, 12902 USF Magnolia Drive, Tampa, FL 33612, USA

**Keywords:** Perirenal hemangioma, Retroperitoneal mass, Vascular neoplasm, Computed tomography, Image-guided biopsy, Renal hilum

## Abstract

Perirenal hemangiomas are rare benign vascular tumors that can present as indeterminate retroperitoneal masses and mimic renal, adrenal, or soft-tissue malignancy. We report the case of a 60-year-old woman who was incidentally found to have a right perirenal mass adjacent to the kidney and adrenal gland. Due to concern for a possible sarcoma, core biopsy was performed and demonstrated a benign vascular neoplasm compatible with hemangioma. This case highlights the diagnostic challenge of rare perirenal hemangiomas and the importance of considering vascular lesions when planning biopsy of enhancing retroperitoneal masses.

## Introduction

Primary retroperitoneal tumors are uncommon, accounting for approximately 0.1%-0.2% of all malignancies; among primary retroperitoneal soft-tissue tumors, an estimated 70%-80% are malignant [1]. The perirenal space contains the kidneys, adrenal glands, renal hilar structures, vessels, lymphatics, and fat, and masses in this compartment may arise from or closely abut several of these structures [2]. Consequently, the organ of origin can be difficult to determine, and the differential diagnosis of an enhancing perirenal mass commonly includes renal cell carcinoma, adrenal neoplasm, paraganglioma, metastatic disease, and retroperitoneal sarcoma.

Hemangiomas are benign vascular neoplasms composed of endothelial-lined vascular channels. They occur most often in the skin, liver, and soft tissues, whereas renal, renal hilar, and perirenal hemangiomas are uncommon. Clinical presentation is variable; perirenal lesions may be detected incidentally or present with nonspecific symptoms related to mass effect or hemorrhage. Imaging features vary with vascular architecture, thrombosis, fibrosis, and fatty or cystic change. On CT or MRI, these lesions may be well circumscribed and demonstrate arterial or peripheral enhancement with persistent delayed enhancement, low T1 signal, and high T2 signal; however, these findings are nonspecific and may closely mimic malignancy [[3], [4], [5], [6], [7], [8]].

Published literature on perirenal hemangiomas consists primarily of isolated case reports and small series. A focused review identified at least 5 well-described perirenal or renal hilar cases closely relevant to the present lesion; these are summarized in Table 1 [[3], [4], [5], [6], [7]]. Because reports use overlapping anatomic labels such as perirenal, renal hilar, and renal capsular, this represents a conservative count of closely comparable cases rather than an exhaustive estimate. These reports illustrate recurrent diagnostic confusion with renal cell carcinoma, liposarcoma, paraganglioma, and metastasis. We report a right perirenal hemangioma that was anatomically separate from the renal parenchyma but supplied by the right renal artery and drained through a renal capsular vein, creating both diagnostic uncertainty and procedural concern.

**Table 1:** Selected published reports of perirenal or renal hilar hemangiomas.

Study	Patient and lesion	Key imaging features/initial impression	Management and diagnosis
Okuno et al. [3]	Female; age not stated in available abstract; 3.5 cm mass at the left renal hilum adjacent to the renal parenchyma	Radiographically difficult to distinguish from renal cell carcinoma	Tumor resection; perirenal hemangioma; no recurrence at 14 mo
Lee et al. [4]	Of 43-y-old woman; right perirenal capillary hemangioma	Peripheral enhancement in the arterial phase with progressive homogeneous enhancement on venous and delayed phases; mild adjacent fat stranding; did not adhere to the kidney	Pathologic examination demonstrated capillary hemangioma surrounded by perirenal fat.
Kishida et al. [7]	Of 75-year-old woman; 26 × 22 × 18 mm mass in the left perirenal space	Well-circumscribed heterogeneous lesion with avid prolonged enhancement, cystic and fatty changes, and surrounding fat stranding; mimicked liposarcoma	Stable for 1 y, then surgically resected; anastomosing hemangioma with fatty change
Jiang et al. [5]	Of 44-y-old woman; 4.8 × 4.0 × 3.2 cm right renal hilar mass	Low T1 and very high T2/DWI signal with marked enhancement and a central hypointense area; initially diagnosed as paraganglioma	Laparoscopic tumor resection with renal preservation; capillary hemangioma
Saldanha et al. [6]	Of 58-y-old man; 2.3 cm right perirenal nodule	Well-circumscribed hypodense lesion with persistent peripheral enhancement and a feeding vessel from the right renal artery; metastasis could not be excluded because of prior melanoma	Laparoscopic kidney-sparing excision; capillary hemangioma
Present case	Of 60-y-old woman; 4.0 × 2.0 × 2.0 cm right perirenal mass	Renal arterial supply, renal capsular venous drainage, near-reniform morphology, and a thin fat plane from the renal parenchyma; differential included sarcoma, paraganglioma, metastasis, and supernumerary kidney	CT-guided core biopsy; benign vascular neoplasm compatible with hemangioma

## Case report

**Fig. 1:**
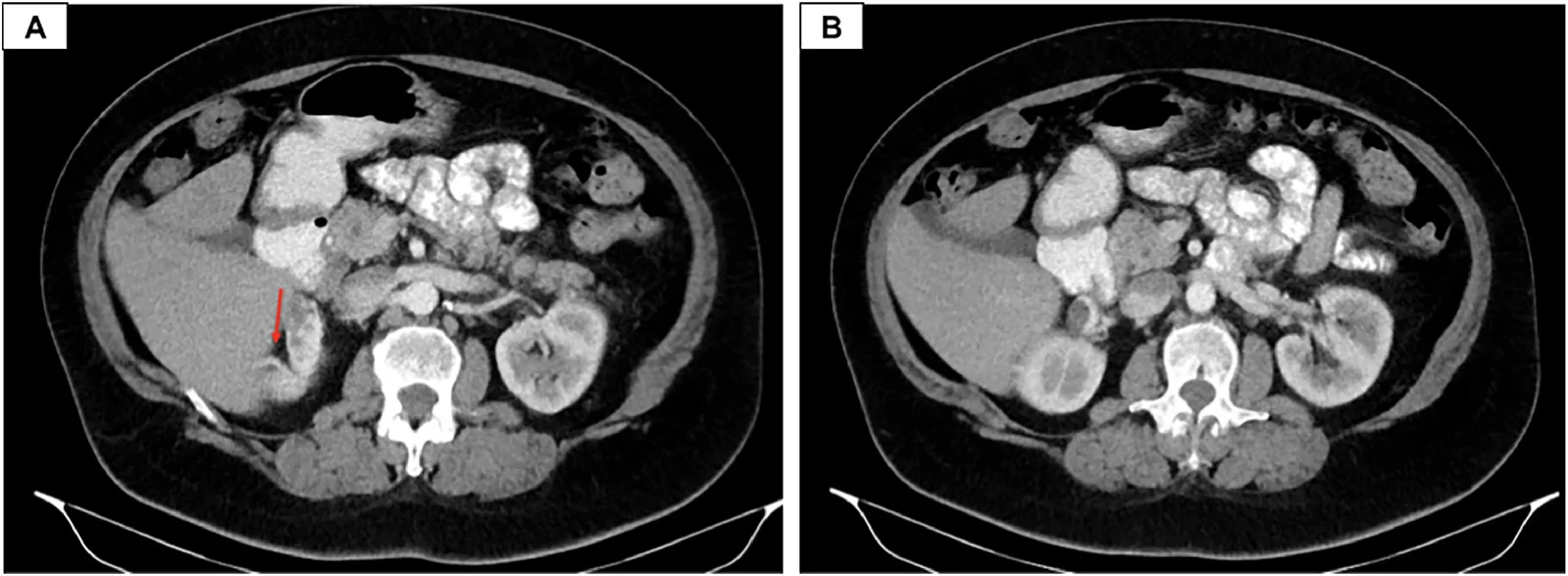
(A) Axial portal venous-phase CT demonstrates the right perirenal lesion; the arrow highlights an associated enhancing renal capsular vein. (B) Follow-up axial CT obtained 9 months after the initial CT demonstrates persistence of the mass adjacent to the right kidney without interval growth.

A 60-year-old woman established care at our institution for continued evaluation of a right retroperitoneal mass and a separate left adrenal nodule. Her workup began approximately 2 years before the current evaluation at an outside hospital, where she presented with severe constipation and poor appetite. No ultrasound was performed during the initial evaluation. CT of the abdomen and pelvis incidentally demonstrated a 4.0 × 2.0 × 2.0 cm heterogeneous right perirenal mass adjacent to the right kidney and adrenal gland (Fig. 1A), as well as a separate 2.0 × 1.5 cm left adrenal nodule. Image-guided biopsy was attempted at the outside hospital but was aborted because no safe percutaneous access window was identified.

The patient was then referred to our institution for evaluation by surgical oncology. At reevaluation, the right-sided lesion remained radiographically indeterminate. Although the patient did not clearly fit the classic clinical profile of pheochromocytoma, paraganglioma remained within the differential diagnosis because of the lesion’s location and enhancement pattern [5,10,11]. Other diagnostic considerations included sarcoma, metastatic disease, and supernumerary kidney. Surgical resection was discussed; however, the patient elected surveillance imaging rather than immediate operative intervention. A follow-up multiphasic CT obtained 9 months after the initial CT demonstrated no interval growth of either lesion (Fig. 1B) and was used to further characterize the right-sided mass. Hormonal laboratory evaluation was also planned for the left adrenal nodule, which had previously appeared most consistent with a benign adenoma [10].

**Fig. 2:**
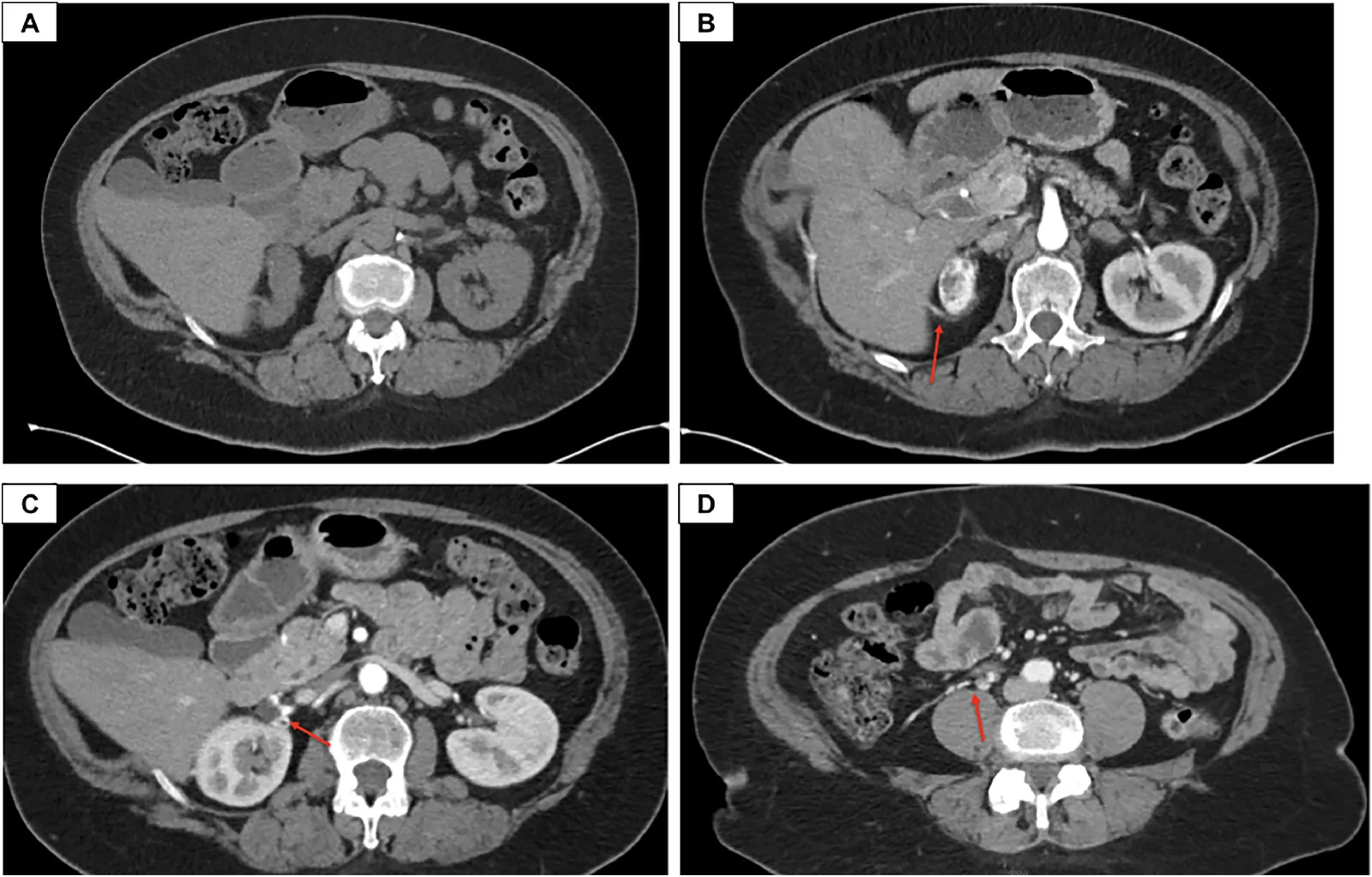
Multiphase CT of the right perirenal mass. (A) Noncontrast CT demonstrates the lesion without internal calcification. (B) Arterial-phase CT demonstrates early enhancement near the right renal hilum and rapid enhancement of the draining vein (arrow). (C) Arterial-phase CT demonstrates arterial supply from the right main renal artery (arrow). (D) Venous-phase CT demonstrates drainage through a renal capsular vein into the right ovarian vein (arrow).

**Fig. 3:**
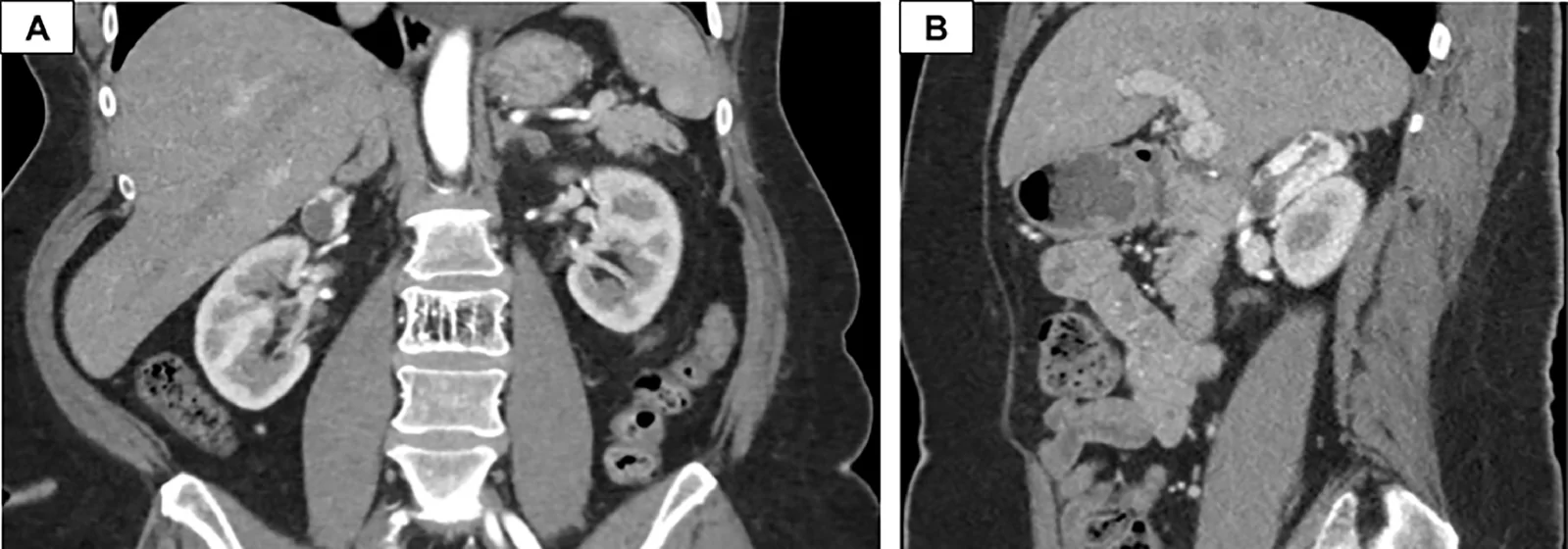
(A) Coronal arterial-phase CT demonstrates the enhancing right perirenal mass closely opposed to the right kidney and adrenal region. (B) Sagittal arterial-phase CT demonstrates a thin intervening fat plane between the lesion and the renal parenchyma, confirming an extrarenal origin.

The 9-month follow-up multiphasic CT demonstrated a persistent right perirenal mass that was anatomically separate from the renal parenchyma but closely associated with the right kidney, adrenal region, and renal hilar structures. Noncontrast, arterial, and venous delayed phases were obtained (Fig. 2). The lesion demonstrated arterial supply from the right renal artery and venous drainage via a renal capsular vein, which ultimately drained into the right ovarian vein (Fig. 2C and D). Coronal and sagittal reconstructions further supported a perirenal location and extrarenal origin (Fig. 3). The lesion closely followed the kidneys in contrast-enhancement characteristics, and its nearly reniform shape on sagittal imaging supported consideration of a supernumerary kidney.

**Fig. 4:**
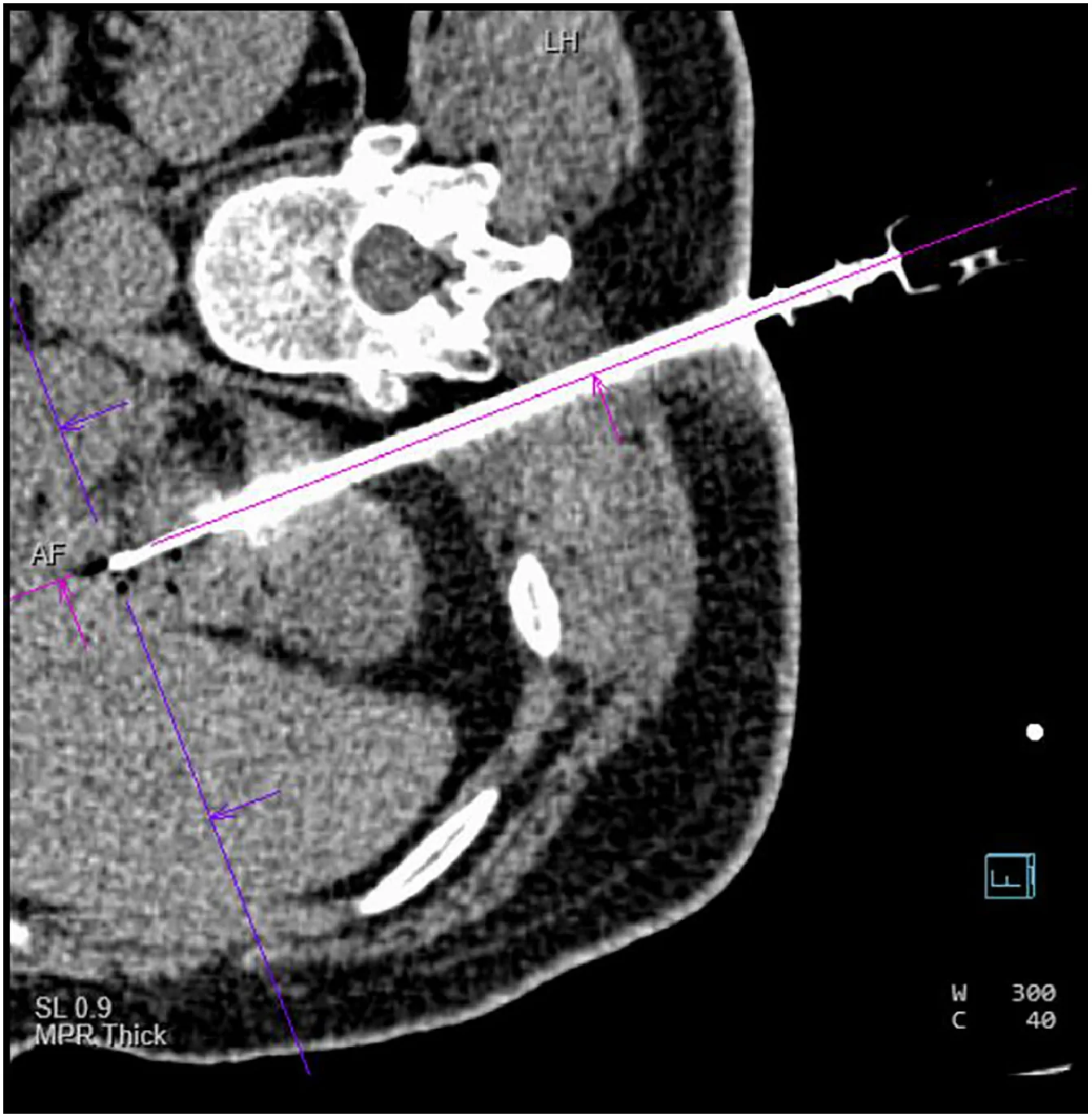
CT-guided biopsy of the right perirenal mass. Intraprocedural CT demonstrates the biopsy needle positioned along a percutaneous paravertebral approach for sampling of the lesion.

Given persistent diagnostic uncertainty and concern for malignancy, percutaneous biopsy was pursued. Consideration was given to prebiopsy angiography to evaluate the arterial supply and allow possible embolization to reduce bleeding risk, but the patient ultimately opted for biopsy with possible embolization if bleeding occurred [[9], [10], [11], [12]]. Intraprocedural CT-guided biopsy demonstrated the biopsy needle positioned along a paravertebral percutaneous approach for sampling of the right perirenal lesion (Fig. 4). A small perilesional hematoma developed during biopsy. Delayed CT imaging showed no interval expansion, so no hemostatic intervention was required. Gelatin sponge (Gelfoam) embolization was available as a contingency and would have been performed if the hematoma had enlarged or active bleeding persisted.

**Fig. 5:**
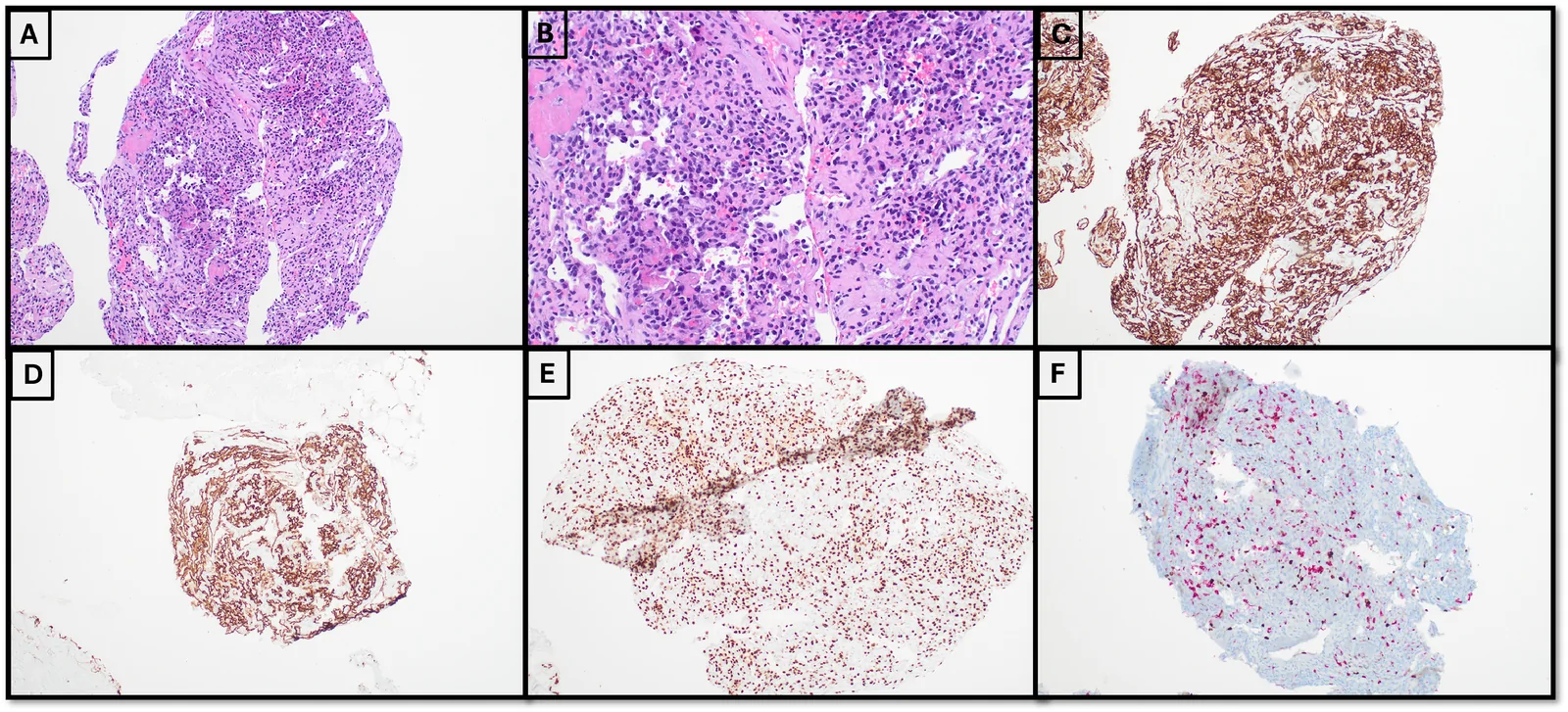
Core biopsy of the right perirenal mass showing a benign vascular neoplasm compatible with hemangioma. (A) H&E, 10× power view, showing a vasoformative lesion. (B) H&E, 20× power view, highlighting bland endothelial-lined vascular spaces without significant atypia or mitotic activity. (C) CD31, 10× power view, demonstrating endothelial positivity. (D) CD34, 10× power view, demonstrating endothelial positivity. (E) ERG, 10× power view, demonstrating nuclear positivity in lesional endothelial cells. (F) Ki-67/CD45 dual stain, 10× power view. The dual stain was performed to assess proliferation within the lesional endothelial component. CD45 highlights admixed hematolymphoid cells, including proliferating cells that could otherwise increase the apparent Ki-67 index, allowing these CD45-positive cells to be excluded from the assessment. The CD45-negative lesional endothelial component demonstrates a low Ki-67 proliferative index (approximately 5%-0%).

Core biopsy of the right perirenal mass demonstrated a benign vascular neoplasm compatible with hemangioma. Histologic examination showed a vasoformative lesion composed of bland endothelial-lined vascular spaces without significant atypia or mitotic activity. Immunohistochemical staining demonstrated endothelial positivity for CD31, CD34, and ERG. No specific histologic subtype was identified on the core biopsy. A Ki-67/CD45 dual stain was performed to more specifically assess proliferation within the lesional endothelial component, as admixed proliferating hematolymphoid cells could otherwise contribute to the apparent Ki-67 index. CD45 highlighted these hematolymphoid cells, allowing them to be distinguished from the CD45-negative endothelial cells and excluded from the assessment. The estimated Ki-67 proliferative index of the lesional endothelial component was low, approximately 5%-10%. The dual stain was used as an adjunct to assess proliferative activity and was not a primary diagnostic criterion for hemangioma. Overall, the morphologic and immunophenotypic findings supported the diagnosis of hemangioma (Fig. 5).

## Discussion

Hemangiomas associated with the kidney have more commonly been described within the renal parenchyma or renal hilum, including capillary and anastomosing subtypes. In contrast, hemangiomas arising entirely within the perirenal space and remaining anatomically separate from the renal parenchyma are exceptionally rare. Because of this location, a perirenal hemangioma may appear to arise from the kidney, adrenal gland, renal hilum, or retroperitoneal soft tissues, creating uncertainty regarding both anatomic origin and malignant potential [[3], [4], [5], [6], [7], [8]].

Published cases demonstrate a recurring pattern of benign vascular lesions mimicking malignant or hormonally active masses. Okuno et al. [3] reported a 3.5 cm left hilar perirenal hemangioma that mimicked renal cell carcinoma; Lee et al. [4] described peripheral arterial enhancement with progressive homogeneous enhancement; Kishida et al. [7] reported an anastomosing hemangioma with fatty change that mimicked liposarcoma; Jiang et al. [5] described a renal hilar capillary hemangioma initially diagnosed as paraganglioma; and Saldanha et al. [6] reported a perirenal capillary hemangioma concerning for melanoma metastasis [[3], [4], [5], [6], [7]]. Table 1 summarizes these selected reports.

These cases highlight a recurring diagnostic problem: vascular enhancement and close association with renal or adrenal structures may suggest malignancy even when the underlying lesion is benign. Multiphasic imaging can help define vascular supply, venous drainage, enhancement kinetics, and the relationship of the lesion to the renal parenchyma, but it may not provide a definitive prebiopsy diagnosis [2,[4], [5], [6], [7], [8]].

The present case adds to this limited literature by emphasizing the importance of accurately characterizing an enhancing perirenal mass as intrarenal or extrarenal. Because the lesion was separable from the renal parenchyma yet closely associated with the right kidney, adrenal region, and renal hilar structures, the radiographic differential reasonably included sarcoma, paraganglioma, metastatic disease, adrenal neoplasm, exophytic renal neoplasm, and supernumerary kidney [5,10,11]. Because imaging did not establish a definitive diagnosis and malignancy remained a concern, percutaneous biopsy was pursued for histopathologic confirmation.

A key practical consideration is biopsy planning. Hypervascular perirenal lesions may carry increased bleeding risk during percutaneous sampling, particularly when located near renal hilar or major retroperitoneal vessels [8,9,12]. When paraganglioma remains within the differential diagnosis, biochemical evaluation is especially important before invasive procedures because biopsy of pheochromocytoma or paraganglioma can be associated with serious complications, including hemorrhage and hypertensive crisis [10,11]. In selected cases, pre-biopsy angiography may help define arterial supply and venous drainage, and embolization may be considered when a dominant feeding vessel is identified [9,12]. In the present case, the small post-biopsy hematoma remained stable on delayed CT, so no intervention was required; gelatin sponge embolization had been planned if the hematoma expanded. For similar hypervascular lesions, strategies to reduce iatrogenic bleeding include careful review of multiphasic imaging to map feeding and draining vessels, selection of a biopsy trajectory that avoids visible vessels and major hilar structures, adherence to periprocedural coagulation and antithrombotic guidelines, and immediate or delayed post-biopsy imaging to identify active bleeding or hematoma expansion [9,12].

Definitive diagnosis in this case relied on histopathologic confirmation. The biopsy demonstrated a benign vasoformative lesion composed of bland endothelial-lined vascular spaces without significant atypia or mitotic activity, and endothelial marker positivity for CD31, CD34, and ERG supported the diagnosis of hemangioma. The Ki-67/CD45 dual stain provided an adjunctive assessment of proliferation by identifying admixed CD45-positive hematolymphoid cells so that their Ki-67 labeling could be excluded from the lesional estimate; the CD45-negative endothelial component showed low proliferative activity (approximately 5%-10%). This low proliferative index supported the indolent nature of the lesion but was not used as a primary diagnostic criterion for hemangioma [13].

Management should be individualized according to symptoms, interval growth, diagnostic certainty, and proximity to major vessels. In the present patient, because biopsy established a benign hemangioma and prior imaging showed no interval growth, no additional lesion-specific follow-up was recommended. For similar cases, observation without dedicated follow-up may be reasonable when pathology is definitive and the patient is asymptomatic; interval imaging can be considered when sampling is limited, diagnostic uncertainty persists, symptoms develop, or growth is suspected. Surgical excision or embolization may be appropriate for symptomatic, enlarging, diagnostically uncertain, or high-risk vascular lesions [6,8]. Improved recognition depends on maintaining vascular tumors in the differential diagnosis, carefully defining vascular anatomy on imaging, and incorporating bleeding risk into biopsy planning.

## Conclusion

This case underscores the diagnostic and procedural challenges posed by an enhancing perirenal mass that is anatomically separate from the renal parenchyma but closely related to the kidney, adrenal region, and renal hilar vasculature. Although initial imaging raised concern for malignant or hormonally active retroperitoneal pathology, histopathologic sampling ultimately confirmed a benign vascular neoplasm compatible with hemangioma. Clinicians should consider vascular lesions in the differential diagnosis of hyperenhancing perirenal or retroperitoneal masses, particularly when imaging demonstrates renal-associated arterial supply or venous drainage. Recognizing this possibility may guide safer biopsy planning, including consideration of angiographic evaluation or embolization in selected cases, while preventing unnecessary surgical resection when a benign diagnosis can be established.

## Declaration of generative AI and AI-assisted technologies in the manuscript preparation process

During the preparation of this work, the authors used ChatGPT to assist with language editing, organization, and manuscript formatting. After using this tool/service, the authors reviewed and edited the content as needed and takes full responsibility for the content of the published article.

## Patient consent

Written informed consent was obtained from the patient for publication of this case report and accompanying images.

## References

[1] Sassa N. (2020). Retroperitoneal tumors: review of diagnosis and management. Int J Urol.

[2] Surabhi V.R., Menias C., Prasad S.R., Patel A.H., Nagar A., Dalrymple N.C. (2008). Neoplastic and non-neoplastic proliferative disorders of the perirenal space: cross-sectional imaging findings. Radiographics.

[3] Okuno T., Ando M., Arisawa C., Okano T. (1999). A case of perirenal hemangioma mimicking renal cell carcinoma. Int J Urol.

[4] Lee J.M., Kim S.W., Kim H.C., Yang D.M., Ryu J.K., Lim S.J. (2016). Dynamic enhanced computed tomographic findings of a perirenal capillary hemangioma. J Korean Soc Radiol.

[5] Jiang W., Liu X., Wen L. (2022). Case report: capillary hemangioma in the renal hilum mimicking paraganglioma. Front Oncol..

[6] Saldanha G., Morgado M., Guerra A. (2024). Perirenal capillary haemangioma: a rare mimicker of malignancy. Eurorad.

[7] Kishida N., Sentani K., Terada H., Honda Y., Goto K., Hatanaka Y. (2017). Anastomosing haemangioma with fatty changes in the perirenal space: a lesion mimicking liposarcoma. BJR Case Rep.

[8] O'Neill A.C., Craig J.W., Silverman S.G., Alencar R.O. (2016). Anastomosing hemangiomas: locations of occurrence, imaging features, and diagnosis with percutaneous biopsy. Abdom Radiol.

[9] Kennedy S.A., Milovanovic L., Midia M. (2015). Major bleeding after percutaneous image-guided biopsies: frequency, predictors, and periprocedural management. Semin Intervent Radiol.

[10] Fassnacht M., Tsagarakis S., Terzolo M., Tabarin A., Sahdev A., Newell-Price J. (2023). European Society of Endocrinology clinical practice guidelines on the management of adrenal incidentalomas, in collaboration with the European Network for the Study of Adrenal Tumors. Eur J Endocrinol.

[11] Vanderveen K.A., Thompson S.M., Callstrom M.R., Young W.F., Grant C.S., Farley D.R. (2009). Biopsy of pheochromocytomas and paragangliomas: potential for disaster. Surgery.

[12] Patel I.J., Rahim S., Davidson J.C., Hanks S.E., Tam A.L., Walker T.G. (2019). Society of Interventional Radiology Consensus Guidelines for the periprocedural management of thrombotic and bleeding risk in patients undergoing percutaneous image-guided interventions-Part II: recommendations: endorsed by the Canadian Association for Interventional Radiology and the Cardiovascular and Interventional Radiological Society of Europe. J Vasc Interv Radiol.

[13] Miettinen M., Wang Z.F., Paetau A., Tan S.H., Dobi A., Srivastava S. (2011). ERG transcription factor as an immunohistochemical marker for vascular endothelial tumors and prostatic carcinoma. Am J Surg Pathol.

